# Blood flow restriction: The acute effects of body tilting and reduced gravity analogues on limb occlusion pressure

**DOI:** 10.1113/EP091874

**Published:** 2024-08-17

**Authors:** Patrick Swain, Nick Caplan, Luke Hughes

**Affiliations:** ^1^ Aerospace Medicine and Rehabilitation Laboratory, Department of Sport, Exercise and Rehabilitation, Faculty of Health and Life Sciences Northumbria University Newcastle upon Tyne UK

**Keywords:** analogue, astronaut, bedrest, blood flow restriction, countermeasure, limb occlusion pressure, microgravity, spaceflight

## Abstract

Blood flow restriction (BFR) has been identified as a potential countermeasure to mitigate physiological deconditioning during spaceflight. Guidelines recommend that tourniquet pressure be prescribed relative to limb occlusion pressure (LOP); however, it is unclear whether body tilting or reduced gravity analogues influence LOP. We examined LOP at the leg and arm during supine bedrest and bodyweight suspension (BWS) at 6° head‐down tilt (HDT), horizontal (0°), and 9.5° head‐up tilt (HUT) positions. Twenty‐seven adults (age, 26 ± 5 years; height, 1.75 ± 0.08 m; body mass, 73 ± 12 kg) completed all tilts during bedrest. A subgroup (*n* = 15) additionally completed the tilts during BWS. In each position, LOP was measured twice in the leg and arm using the Delfi Personalized Tourniquet System after 5 min of rest and again after a further 5 min. The LOP at the leg increased significantly from 6° HDT to 9.5° HUT in bedrest and BWS by 9–15 mmHg (Cohen's *d* = 0.7–1.0). Leg LOP was significantly higher during BWS at horizontal and 9.5° HUT postures relative to the same angles during bedrest by 8 mmHg (Cohen's *d* = 0.6). Arm LOP remained unchanged between body tilts and analogues. Intraclass correlation coefficients for LOP measurements taken after an initial and subsequent 5 min rest period in all conditions ranged between 0.91–0.95 (leg) and 0.83–0.96 (arm). It is advised that LOP be measured before the application of a vascular occlusion in the same body tilt/setting to which it is applied to minimize discrepancies between the actual and prescribed tourniquet pressure.

## Abstract (highlights)

- **What is the central question of this study?**
  Application of blood flow restriction (BFR) requires tourniquet cuff pressure to be prescribed on an individual basis relative to limb occlusion pressure (LOP). It is unknown how body tilting or reduced gravitational environments influence LOP measurement.
- **What is the main finding and its importance?**
  Leg LOP increased significantly from 6° head‐down (microgravity analogue) tilt to 9.5° head‐up tilt (lunar gravity analogue) in bedrest and bodyweight suspension analogues and was significantly higher during bodyweight suspension versus bedrest at horizontal and 9.5° head‐up tilt angles. The findings suggest that body tilting and reduced gravitational environments could affect LOP measurement, which has implications for the use of BFR in future space exploration missions.

## 1 INTRODUCTION

Astronauts experience a wide range of adaptations upon entering the spaceflight environment, including musculoskeletal and cardiovascular deconditioning, impaired sensorimotor performance, orthostatic intolerance, altered immune response and spaceflight‐associated neuro‐ocular syndrome (Crucian et al., 2018; Jirak et al., 2022; Lee et al., 2020; Qaisar et al., 2020; Stavnichuk et al., 2020; Vernice et al., 2020). Spaceflight‐induced deconditioning presents many risks to astronaut health and performance on mission and upon return to Earth (NASA, n.d.). Space agencies have implemented spaceflight countermeasure programs to attenuate the adverse physiological effects of spaceflight, with exercise (aerobic and resistive) being one of the primary countermeasures (Korth, 2015; Loerch, 2015). The current exercise countermeasure program used on the International Space Station (ISS) is, however, time‐consuming, with astronauts allotted up to ∼2.5 h a day, 6 days a week, for exercise‐related activities, and there remain concerns regarding its effectiveness (Hackney et al., 2015; Loerch, 2015; Scott et al., 2019a). In addition, the ISS exercise hardware (treadmill, cycle ergometer, and a specialized resistive system) is too large to be accommodated in future space exploration mission scenarios (e.g., to the moon), where astronauts will transit to a considerably smaller lunar orbiting space station (Gateway) in a capsular vehicle (e.g., NASA's Orion capsule) (Laws et al., 2020). There is an ongoing search for an ‘all‐in‐one’ countermeasure that can meet the technical constraints of exercising in small spacecraft/stations and effectively maintain astronaut health and performance within fitness for duty standards using as little time (for exercise) and mission resources as possible (NASA, 2022; Scott et al., 2019a, 2019b).

Blood flow restriction (BFR) exercise has been identified as a potential countermeasure against spaceflight‐induced deconditioning of the musculoskeletal and cardiovascular systems (Behringer & Willberg, 2019; Hackney et al., 2012; Hughes et al., 2022; Willis et al., 2019). In brief, BFR exercise involves an application of a tourniquet cuff on the proximal leg(s)/arm(s) that is subsequently inflated to a predetermined pressure to compress the underlying vasculature with the aim of causing a partial arterial and complete venous occlusion in the exercising limb (Hughes, Rosenblatt, et al., 2018). A unique feature of BFR exercise is that it can allow users to exercise with low mechanical loads (20%–40% of one‐repetition maximum; <50% of maximal aerobic capacity) whilst eliciting positive training adaptations in muscular and cardiovascular tissues alongside various performance outcomes (Hughes, Rosenblatt, et al., 2018; Silva et al., 2019). Emerging evidence also suggests that BFR might provide an osteogenic stimulus for bone adaptation (Hughes & Centner, 2024). Comprehensive reviews on the acute and chronic effects of BFR exercise and mechanistic theories underlying these responses can be found elsewhere (Hughes et al., 2022; Jessee et al., 2018; Silva et al., 2019).

It is currently recommended that tourniquet pressure be applied on an individual basis at 40%–80% of the user's limb occlusion pressure (LOP; the minimum pressure required to completely occlude arterial blood flow in the limb completely for a specific tourniquet and user) (McEwen et al., 2019b; Patterson et al., 2019). The LOP varies between individuals; a large cohort study identified that limb girth and blood pressure account for approximately 50%–60% of interindividual variability in arm and leg LOP (Loenneke et al., 2015). Practices whereby tourniquet pressure is prescribed using fixed arbitrary pressures (e.g., 200 mmHg) or at a given percentage of systolic blood pressure, therefore, will impose varying degrees of arterial occlusion within a group of individuals that could affect safety, efficacy, and user tolerability (McEwen et al., 2019a; Murray et al., 2021). Beyond BFR exercise, LOP also serves as a valuable measurement in various other contexts, including ischaemic preconditioning, assessment of vascular status, perioperative planning (e.g., collateral circulation, risk of ischaemic complications and wound healing), surgery (creating a bloodless surgical field for extremity operations) and aiding in diagnostic testing (e.g., peripheral arterial disease).

A new line of research has been examining the effect of body posture on LOP (Hughes, Jeffries, et al., 2018; Karanasios et al., 2022; Sieljacks et al., 2017). It has been observed that within an individual, LOP at the upper leg differs significantly between lying (187 ± 33 mmHg), sitting (204 ± 29 mmHg), and standing (242 ± 49 mmHg) postures (Hughes, Jeffries, et al., 2018). A separate study observed LOP in the upper arm to change by ∼5–14 mmHg between the same postures, indicating that arm LOP is less sensitive to postural changes (Karanasios et al., 2022). The implications of LOP being affected by posture are significant because it implies that the application of vascular occlusion in a posture different from that to which LOP was measured can lead to discrepancies between the target and actual percentage of LOP applied. Two main issues arise from this situation: (1) misapplication of excessively high tourniquet pressures; or (2) use of suboptimal/ineffective pressures. Knowing that LOP changes rapidly in response to body posture (standing, sitting, and lying) suggests that there are acute factors that can explain intraindividual variability in LOP and might be related to altered haemodynamics, fluid redistributions, autonomic activity and/or muscle contractile status (Hughes, Jeffries, et al., 2018).

In the context of astronauts using BFR as a countermeasure in future space exploration missions, it is not known whether LOP is affected by reduced gravitational environments that are known to cause a plethora of short‐ and long‐term effects within almost all physiological systems (Jirak et al., 2022; Lee et al., 2022; Mulavara et al., 2018; Stower, 2019; Vernice et al., 2020; Vico & Hargens, 2018). Given that LOP is measured by subjecting the limb to increasing occlusion pressures and evaluating changes in blood flow (via Doppler ultrasound) or arterial pressure pulsations (via automated tourniquet cuffs), it is plausible that LOP might also exhibit sensitivity to body tilting even when the posture of the body remains unchanged. There are a multitude of spaceflight‐induced responses that could be suggested to influence an astronaut's LOP, including equalization of hydrostatic pressure gradients across the body, cephalad fluid shifts, deconditioning of muscle, cardiac and vascular tissues, altered limb circumference (a major predictor of LOP), altered haemodynamics (increased stroke volume and cardiac output and reduced systemic vascular resistance), loss of plasma volume, and increased sympathetic activity coupled with (paradoxically) vasodilatation (Austin et al., 2014; Hargens et al., 2013; Jirak et al., 2022; Lane et al., 1997; Moore & Thornton, 1987; Norsk, 2020; Norsk & Christensen, 2009; Norsk et al., 2015).

Body tilting in head‐up and head‐down positions provides a useful means with which to gain insight into the effect of manipulating the gravitational acceleration vector along the longitudinal axis of the body on LOP. Whittle et al. (2022) established through tilting individuals up to ±45° that the cardiovascular system is highly responsive to its gravitational environment and observed haemodynamic and autonomic ‘gravitational dose–response curves’. The internationally preferred analogue for simulating microgravity is 6° head‐down tilt (HDT), because this tilt mimics key physiological responses reported in astronauts during spaceflight, particularly the acute shift in body fluids from the lower limbs towards the thorax and head (Smith et al., 2011). Furthermore, 9.5° head‐up tilt (HUT) has been used as a lunar gravity analogue, allowing the body to experience a 0.17*g* (1.62 m s^−2^) acceleration along the longitudinal axis (Cavanagh et al., 2013). Astronauts would experience ∼17% bodyweight loading on the lunar surface; therefore, to replicate this environment in a laboratory setting more accurately, bodyweight suspension (BWS) analogues have been devised. Head‐up tilt BWS operates by suspending the body in a 9.5° HUT position by a series of limb support slings to allow for a component of gravity to act along the longitudinal axis, effectively accelerating the user onto a foot platform at a desired level of weight‐bearing as a function of the HUT angle [*mg* × sin(α), where m = body mass, *g* = gravitational acceleration (9.81 m s^−2^, and α = head‐up tilt angle]. Examining LOP across these different body tilts/analogues would provide new insight into whether body tilting, independent of posture, can influence LOP, and has implications for the application of a vascular occlusion to a user in a tilted position and astronauts within reduced gravitational environments.

In the present study, we examined the acute effects and reliability of LOP at the leg and arm during bedrest and BWS at 6° HDT (simulated microgravity), 0° tilt (horizontal reference posture) and 9.5° HUT (simulated lunar gravity, by allowing ∼17% bodyweight loading). In addition, we also performed an exploratory analysis on whether LOP differed between bedrest and BWS analogues at the same body tilt, which has implications for the transferability of LOP between alike settings.

It was hypothesized that: (1) LOP would significantly increase at the leg and decrease at the arm in the following order: 6° HDT → 0° tilt → 9.5° HUT, owing to body fluid redistributions; (2) LOP would not be significantly different between BWS and bedrest conditions at the same body tilt; and (3) LOP would have excellent test–retest reliability in the arm and leg in all experimental conditions, with expected intraclass correlation coefficients (ICCs) of >0.85 based on previous research (Hughes, Jeffries, et al., 2018; Karanasios et al., 2022).

## 2 METHODS

### 2.1 Ethical approval

All participants provided written informed consent prior to participation. The study conformed to the standards set by the 2013 *Declaration of Helsinki*, except for registration in a database, and received local ethical approval by the Northumbria University Institutional Review Board (submission reference: 46356).

### 2.2 Participants

Twenty‐seven adults (*n* = 20 males; *n* = 7 females) participated in the study; participant characteristics are displayed in Table 1. All participants were self‐confirmed not to suffer from orthostatic hypotension or any medical condition/prescription that could potentially influence the study outcomes and were not known to be pregnant. Participant body mass and height were recorded with a calibrated Seca 703 digital scale (to the nearest 0.1 kg) and Seca 213 stadiometer (to the nearest 0.01 m), respectively. Blood pressure was measured on the left arm using an automated cuff (UA‐611 Automatic Blood Pressure Monitor, A&D Medical). The circumferences of the thigh and arm were measured to the nearest centimeter using a flexible anthropometric tape measure (Diameter Tape W606PD, Lufkin).

**TABLE 1:** Participant characteristics.

Characteristic	Study cohort (*n* = 27)
Male:female, *n*	20:7
Age, years	26 ± 5
Height, m	1.75 ± 0.08
Body mass, kg	73 ± 12
Systolic blood pressure, mmHg	125 ± 10
Diastolic blood pressure, mmHg	72 ± 9
Resting heart rate, beats min^−1^	61 ± 11
Thigh circumference, cm	55 ± 4
Arm circumference, cm	30 ± 3

*Note*: Values are the mean ± SD.

Sample size requirements were estimated from several statistical power analyses (G*power, v.3.1) using data from previous studies investigating posturally induced changes in leg LOP between supine lying, sitting and/or standing positions (Hughes, Jeffries, et al., 2018; Sieljacks et al., 2017). Leg LOP was selected as the primary outcome of interest for the power analyses because BFR is more commonly applied for lower‐body exercise, and the lower limbs of astronauts are preferentially affected by spaceflight compared with the upper limbs (Tanaka et al., 2017). Samples of *n* = 8, 13 and 28 were estimated (α = 0.05, β = 81%–86%) based on pairwise comparisons using the Cohen's *d* effect size (ES) statistic between supine lying, sitting and standing conditions (ES = 0.55–1.25) (Hughes, Jeffries, et al., 2018). Samples of *n* = 6 and 19 (α = 0.05, β = 82%–83%) were estimated based on ESs between supine lying versus sitting using wide and narrow BFR cuffs (ES = 0.70–1.50) (Sieljacks et al., 2017). To reflect the present study design, an additional power analysis was performed using a 1 × 3 model (one group × three repeated measures) with an assumed partial eta squared of 0.06 (Cohen's *f* = 0.25) used as a threshold for a medium effect and yielded a required sample of *n* = 27 (α = 0.05, β = 80%, correlation among repeated measures = 0.5).

### 2.3 Study design

Participants visited the laboratory on one occasion during standard working hours (09.00–17.00 h). All participants completed the following bedrest body tilt conditions in a randomized order: 6° HDT, horizontal (0° tilt) and 9.5° HUT. A subgroup of participants (*n* = 15) also completed the same body tilts (order randomized) in a BWS microgravity and hypogravity analogue. Bedrest and BWS tests were performed in a counterbalanced order among these participants. For each body tilt condition, participants rested for 5 min, after which two LOP measurements were taken at the leg and arm and averaged at each site for analysis. A second set of LOP measurements were taken after a further 5 min of rest to determine within‐session reliability. The side of the body (left or right) on which the tourniquet cuff was placed to measure LOP was randomized for each participant. The first limb for which LOP was measured (leg or arm) was randomized for each participant, and the order of measurements was performed as follows: limb A → limb B → limb A → limb B. Participants were instructed to remain hydrated, eat a standard balanced meal ≥2 h before attending the laboratory, wear loose shorts and a t‐shirt, and avoid stimulant ingestion (e.g., caffeine), alcohol and vigorous exercise on the day of testing. All tests were conducted in a controlled laboratory environment (temperature, 20.0°C ± 0.9°C; humidity, 51% ± 11%; barometric pressure, 1023 ± 23 hPa).

### 2.4 Bedrest

Participants laid in a supine position on a medical plinth with an in‐built electric tilting mechanism (Medi‐Plinth 3‐Section Electric Plinth, Linak, UK). The plinth was tilted to the target body tilt angle using a digital inclinometer (RS PRO 175 mm LCD, RS Components, London, UK) to the nearest 0.1° before each test. In the horizontal position, the axial (head‐to‐toe) gravity vector equates to 0*g*, with Earth's gravity vector acting in the anterior–posterior direction. When oriented to a 9.5° HUT, the component of Earth's gravity acting in the axial direction equates to 0.17*g* [*mg* × sin(*α*), where m = body mass, *g* = gravitational acceleration (9.81 m s^−2^), and α = head‐up tilt angle], simulating lunar gravity. There was no foot platform at the end of the bed, thus, during simulated lunar gravity (9.5° HUT), participants remained non‐weight‐bearing at the feet. A 6° HDT orientation is an internationally recognized analogue for microgravity, frequently used in space agency‐sponsored long‐duration bedrest studies (Hargens & Vico, 2016). Although this leads to a negative axial gravity vector, 6° HDT elicits similar cephalic fluid shifts in the body to those seen in a real microgravity environment (Hargens & Vico, 2016).

### 2.5 Bodyweight suspension

**FIGURE 1:**
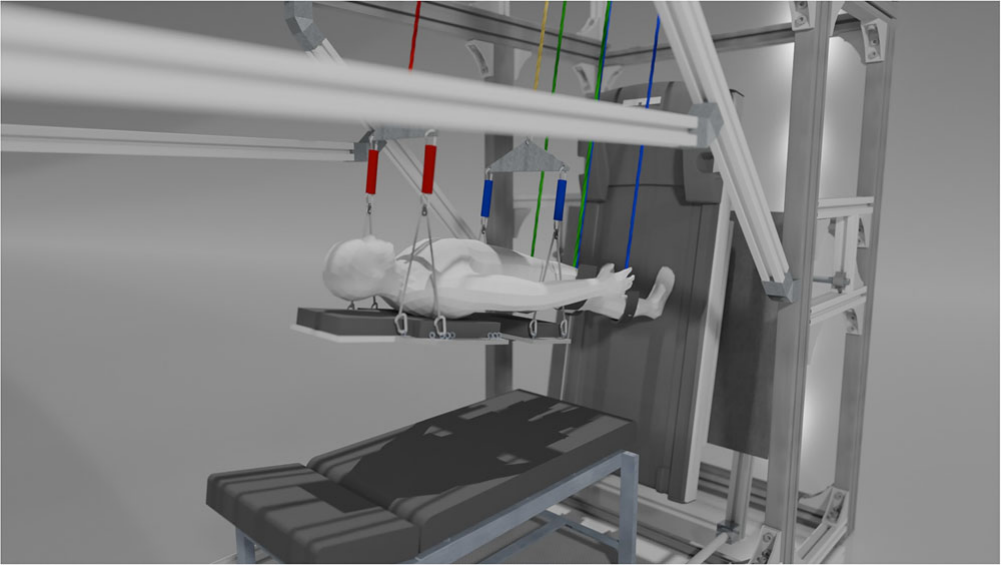
Photorealistic three‐dimensional model (created using SketchUp software) of a user standing in simulated lunar gravity (9.5° head‐up tilt) within the Variable Gravity Suspension System (VGSS).

Bodyweight suspension tests were performed using the Variable Gravity Suspension System (VGSS), a bespoke designed whole‐body suspension system that facilitates both micro‐ and hypo‐gravity analogue conditions (Figure 1). The suspension procedure was as follows: (1) participants laid upon a custom pelvic‐to‐head back support plate situated on top of the medical plinth tilted to the target tilt angle; (2) slings were then secured around the lower limbs; (3) limb/support plate slings were connected to an overhanging rope system and rope slack was removed; and (4) the medical plinth was lowered, suspending the participant midair at the target body tilt. Participants suspended in the 6° HDT and 0° tilt positions remained non‐weight‐bearing. In the 9.5° HUT position, a component of Earth's gravity acts in the head‐to‐toe direction, accelerating the body towards the VGSS foot platform, allowing the participant to stand in simulated lunar gravity (0.17*g*). A force plate (AMTI, MA, USA) mounted onto the VGSS foot platform verified vertical ground reaction forces of the participant during lunar gravity simulations throughout a 30 s period at 2000 Hz. A low‐pass 20 Hz second‐order Butterworth filter was applied to reduce signal noise. Participants experienced 19.6% ± 1.9% weight‐bearing in lunar gravity (9.5° HUT) simulations (range, 17.1%–23.6%).

### 2.6 Limb occlusion pressure

Limb occlusion pressure was measured in the leg and arm using an automatic personalized tourniquet system (Delfi Medical Innovations, Vancouver, BC, Canada). This system comprised a dual‐purpose tourniquet cuff (11.5 cm × 86 cm) connected to a pneumatic device via an airtight hose tubing. This system has been validated against Doppler ultrasound and distal photoplethysmography techniques for measuring LOP (Hughes & McEwen, 2021; Masri et al., 2016) and is reliable on a test–retest basis, with reported ICCs of 0.95–0.98 (Hughes, Jeffries, et al., 2018). Participants wore limb‐protection sleeves on the proximal section of the upper leg and arm, on top of which the tourniquet cuff was secured via Velcro straps. Participants were familiarized with the LOP measurement in both limbs at the beginning of the session.

### 2.7 Statistical analysis

The Statistical Package for Social Sciences (SPSS, v.29, IBM Corporation) was used for statistical analyses. Differences in LOP after 5 min of rest at each body tilt within the bedrest and (independently) BWS were compared using a one‐way repeated‐measures ANOVA. The LOP data for the leg and arm were normally distributed in all conditions according to the Shapiro–Wilk test (*P* > 0.05), except for leg LOP at 6° HDT during bedrest (*P* = 0.04). Further inspection of leg LOP at 6° HDT during bedrest revealed trivial skewness (<±0.5) and acceptable kurtosis (<±2.0) coupled with the (normal) *Q–Q* plot indicating no major deviations in the observed data from the reference line (George, 2011). The ANOVA is considered generally robust against violations of normality; therefore, no data transformations were used (Caldwell et al., 2022). Mauchly's test of sphericity indicated a violation of sphericity (ε = 0.81; *P* = 0.03) for within‐participant effects between the bedrest body tilts for leg LOP; therefore, the degrees of freedom and *P*‐value of the ANOVA were interpreted using the Greenhouse–Geisser correction. Sphericity was met (*P* > 0.05) for all other ANOVAs. Pairwise comparisons between body tilts were assessed using Bonferroni *post hoc* adjustments. For the subgroup (*n *= 15) that completed both bedrest and BWS, Student's paired *t*‐tests were used to compare LOP at the same body tilt between analogues.

Intraclass correlation coefficients with 95% confidence intervals (CIs) were used to determine within‐session test–retest reliability for the two leg and arm LOP measurements between the first and second 5 min rest period for each body tilt condition. The ICCs were modelled using single measures (type) and two‐way mixed effects (model) with absolute agreement (definition) (Koo & Li, 2016). Reliability scores were classified via the following ICC thresholds: <0.50 (poor), 0.50–0.74 (moderate), 0.75–0.89 (good) and ≥0.90 (excellent) (Koo & Li, 2016). The within‐participant coefficient of variation (CV%) was also calculated for each condition in accordance with previous research, whereby the standard deviation (SD) of the two LOP measurements is divided by their corresponding mean and multiplied by 100 to yield a percentage (Hughes, Jeffries, et al., 2018). Standardized mean differences (SMDs) between trials were calculated using Cohen's *d* ES and classified according to the following qualitative descriptors: 0.2 (small), 0.5 (medium), 0.8 (large) and 1.3 (very large) (Rosenthal, 1996). Statistical significance was set at *P* < 0.05. Data are presented as the mean ± SD.

## 3 RESULTS

All participants completed the bedrest trials (*n* = 27), and a subset of these participants (*n* = 15) also completed the BWS trials. No adverse events were reported among ∼1000 LOP measurements. The LOP was measured successfully in ∼97% of cases. A total of 33 LOP recordings (∼3%) were not obtained owing to a ‘noisy signal error’ after three consecutive measurement attempts. If at least one LOP measurement was successful at the given body tilt and time point (27 of 33 cases), the singular LOP value was used in lieu of an average of two measurements. In the remaining six cases, both LOP measurements were unable to be obtained for the given tilt and time point and were excluded from analyses.

### 3.1 Effect of body tilt on LOP

**FIGURE 2:**
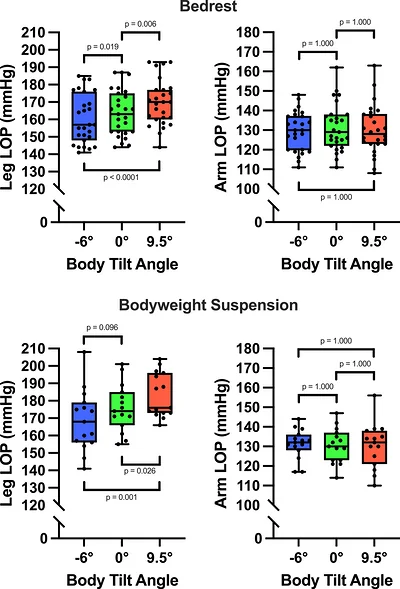
Boxplots for leg and arm limb occlusion pressure (LOP) during bedrest and bodyweight suspension at 6° head‐down tilt (HDT), 0° tilt (horizontal) and 9.5° head‐up tilt (HUT) body tilts. Bedrest plots are *n* = 27, except for arm LOP at 6° HDT and 9.5° HUT, for which *n* = 26. Bodyweight suspension plots are *n* = 15. Boxplots display the median (horizontal line), 25th–75th percentiles (box boarders) and the minima and maxima (error bars). Individual values are displayed as filled circles.

Table 2 presents leg and arm LOP during 6° HDT, 0° tilt and 9.5° HUT in bedrest and BWS conditions. Leg LOP revealed a significant within‐participant effect of tilt angle during bedrest (*F*_1.62,42.03_ = 21.860, *P* < 0.0001) and BWS (*F*_2,28_ = 12.880, *P* < 0.0001) (Figure 2); pairwise comparisons are presented in Table 3. Arm LOP showed no significant within‐participant effect between body tilts during bedrest (*F*_2,50_ = 0.322, *P* = 0.726) or BWS (*F*_2,28_ = 0.149, *P* = 0.862) (Figure 2); pairwise comparisons are presented in Table 4.

**TABLE 2:** Leg and arm limb occlusion pressure between body tilt conditions in bedrest and bodyweight suspension.

Condition	Leg LOP (mmHg)		Arm LOP (mmHg)	
	Bedrest	Suspension	Bedrest	Suspension
6° HDT	160 ± 14	168 ± 18	129 ± 10	131 ± 7
0° Tilt	163 ± 13	176 ± 14	131 ± 12	130 ± 9
9.5° HUT	169 ± 13	183 ± 13	130 ± 13	130 ± 11

*Note*: Values are the mean ± SD. These data reflect the first set of LOP measurements taken after 5 min of rest in each condition. *n* = 27 for all bedrest results, except for arm LOP in 6° HDT and 9.5° HUT (*n* = 26) owing to missing data. *n* = 15 for all suspension results.

Abbreviations: HDT, head‐down tilt; HUT, head‐up tilt; LOP, limb occlusion pressure.

**TABLE 3:** Bonferroni pairwise comparisons for leg limb occlusion pressure between body tilts during bedrest and bodyweight suspension.

Condition	Pairwise comparison	LOP mean difference (95% CI) (mmHg)	Cohen's *d*	*P*‐value
Bedrest (*n* = 27)				
0° Tilt vs.	6° HDT	3 (0, 6)	0.22	0.019
	9.5° HUT	−6 (−10, −2)	0.46	0.006
6° HDT vs.	0° Tilt	−3 (−6, 0)	0.22	0.019
	9.5° HUT	−9 (−13, −6)	0.67	<0.0001
9.5° HUT vs.	0° Tilt	6 (2, 10)	0.46	0.006
	6° HDT	9 (6, 13)	0.67	<0.0001
Bodyweight suspension (*n* = 15)				
0° Tilt vs.	6° HDT	7 (−1, 16)	0.37	0.096
	9.5° HUT	−7 (−14, −1)	0.50	0.026
6° HDT vs.	0° Tilt	−7 (−16, 1)	0.37	0.096
	9.5° HUT	−15 (−23, −6)	0.96	0.001
9.5° HUT vs.	0° Tilt	7 (1, 14)	0.50	0.026
	6° HDT	15 (6, 23)	0.96	0.001

Abbreviations: HDT, head‐down tilt; HUT, head‐up tilt; LOP, limb occlusion pressure.

**TABLE 4:** Bonferroni pairwise comparisons for arm limb occlusion pressure between body tilts during bedrest and bodyweight suspension.

Condition	Pairwise comparison	LOP mean difference (95% CI) (mmHg)	Cohen's *d*	*P*‐value
Bedrest (*n* = 26)				
0° Tilt vs.	6° HDT	1 (−2, 5)	0.18	1.000
	9.5° HUT	1 (−2, 3)	0.08	1.000
6° HDT vs.	0° Tilt	−1 (−5, 2)	0.18	1.000
	9.5° HUT	0 (−4, 3)	0.09	1.000
9.5° HUT vs.	0° Tilt	−1 (−3, 2)	0.08	1.000
	6° HDT	0 (−3, 4)	0.09	1.000
Bodyweight suspension (*n* = 15)				
0° Tilt vs.	6° HDT	−1 (−6, 4)	0.12	1.000
	9.5° HUT	0 (−6, 5)	0.00	1.000
6° HDT vs.	0° Tilt	1 (−4, 6)	0.12	1.000
	9.5° HUT	1 (−4, 6)	0.11	1.000
9.5° HUT vs.	0° Tilt	0 (−5, 6)	0.00	1.000
	6° HDT	−1 (−6, 4)	0.11	1.000

Abbreviations: HDT, head‐down tilt; HUT, head‐up tilt; LOP, limb occlusion pressure.

### 3.2 Effect of bedrest and bodyweight suspension on LOP

**FIGURE 3:**
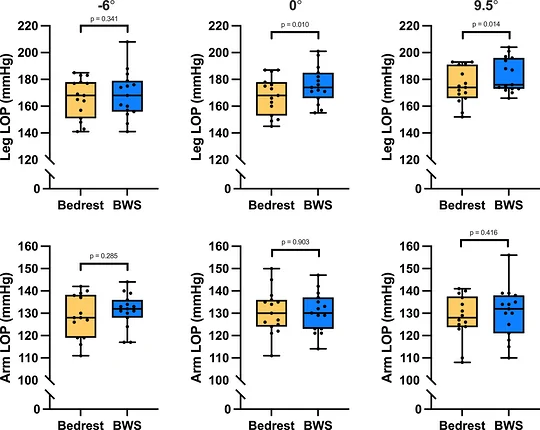
Boxplots for limb occlusion pressure (LOP) at the leg (top plots) and arm (bottom plots) between bedrest and bodyweight suspension (BWS) analogues at 6° head‐down tilt (HDT), horizontal 0° tilt and 9.5° head‐up tilt (HUT). All are *n* = 15, except for arm LOP at 6° HDT and 9.5° HUT, for which *n* = 14. Boxplots display the median (horizontal line), 25th–75th percentiles (box boarders) and the minima and maxima (error bars). Individual values are displayed as filled circles.

Leg and arm LOP in bedrest and BWS analogues at the same body tilt for the subgroup of participants (*n* = 15) that completed both conditions are displayed in Figure 3. Leg LOP was significantly higher during BWS compared with bedrest at 0° tilt (*t*_14_ = −2.955, *P* = 0.010) by 8 mmHg (95% CI: −2, 14 mmHg; SMD = 0.58) and 9.5° HUT (*t*_14_ = −2.801, *P* = 0.014) also by 8 mmHg (95% CI: −2, 14 mmHg; SMD = 0.61). Leg LOP was not significantly different between bedrest and BWS at 6° HDT (*t*_14_ = −0.990, *P* = 0.341), where the mean change was 3 mmHg (95% CI: −3, 8 mmHg; SMD = 0.16). Arm LOP was not significantly different between BWS and bedrest in any of the body tilts: 0° tilt (*t*_14_ = 0.125, *P* = 0.903), 6° HDT (*t*_13_ = −1.116, *P* = 0.285) and 9.5° HUT (*t*_13_ = −0.841, *P* = 0.416), with mean differences of 0 mmHg (95% CI: 2, −4 mmHg; SMD = 0.03), 2 mmHg (95% CI: −2, 6 mmHg; SMD = 0.24) and 1 mmHg (95% CI: −3, 6 mmHg; SMD = 0.16), respectively (Figure 3).

### 3.3 Reliability of LOP

**FIGURE 4:**
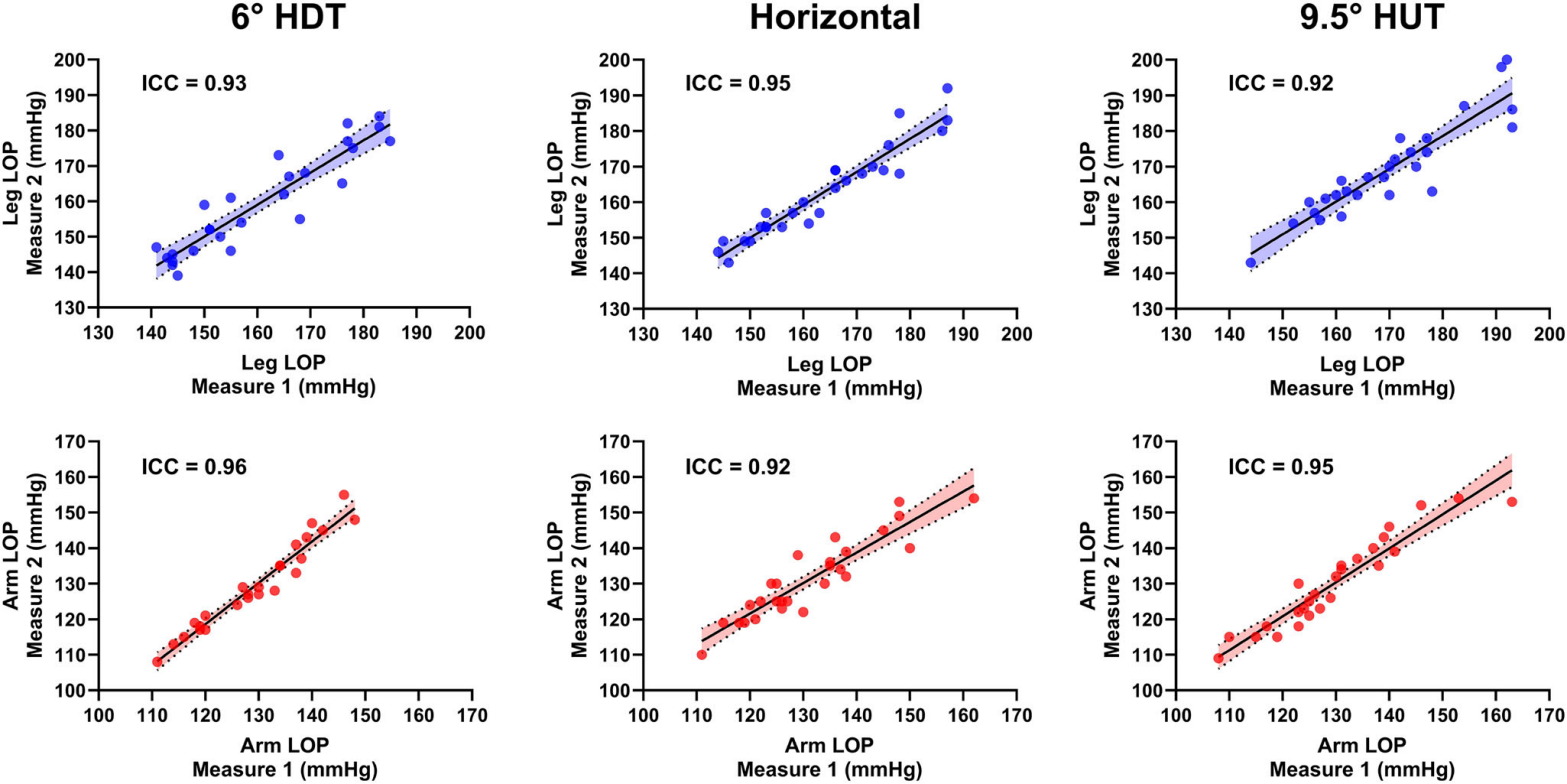
Reliability of limb occlusion pressure (LOP) measured at the leg (top plots) and arm (bottom plots) following two consecutive 5 min periods of rest during bedrest in the 6° head‐down tilt (HDT), 0° tilt (horizontal) and 9.5° head‐up tilt (HUT) conditions. Data are fitted with linear regression lines and 95% confidence interval bands. All ICCs are *P* < 0.0001. Abbreviation: ICC, intraclass correlation coefficient.

Limb occlusion pressure measurements at the leg and arm following two consecutive 5 min rest periods at each body tilt had ICCs ranging between 0.91 and 0.95 and between 0.83 and 0.96, respectively (Figure 4; Table 5). The mean within‐participant CV% was 1%–2% for all test–retest LOP measurements.

**TABLE 5:** Test retest reliability of leg and arm limb occlusion pressure at each body tilt during bedrest and bodyweight suspension.

Condition	LOP (mmHg)		ICC (95% CI)	CV%
	Measurement 1	Measurement 2		
**Leg**				
Bed 6° HDT (*n* = 26)	160 ± 14	159 ± 14	0.93 (0.84, 0.97)*	1.85 ± 1.65
Bed 0° Tilt (*n* = 27)	163 ± 13	162 ± 13	0.95 (0.90, 0.98)*	1.36 ± 1.05
Bed 9.5° HUT (*n* = 27)	169 ± 13	169 ± 13	0.92 (0.83, 0.96)*	1.63 ± 1.43
BWS 6° HDT (*n* = 15)	168 ± 18	170 ± 13	0.91 (0.75, 0.97)*	2.16 ± 1.84
BWS 0° Tilt (*n* = 15)	176 ± 14	173 ± 16	0.92 (0.78, 0.97)*	2.08 ± 1.49
BWS 9.5° HUT (*n* = 15)	183 ± 13	185 ± 12	0.91 (0.76, 0.97)*	1.44 ± 1.33
Arm				
Bed 6° HDT (*n* = 25)	129 ± 10	130 ± 13	0.96 (0.91, 0.98)*	1.30 ± 1.06
Bed 0° Tilt (*n* = 27)	131 ± 12	130 ± 11	0.92 (0.84, 0.96)*	1.91 ± 1.51
Bed 9.5° HUT (*n* = 26)	130 ± 13	130 ± 13	0.95 (0.89, 0.98)*	1.67 ± 1.17
BWS 6° HDT (*n* = 14)	131 ± 7	132 ± 9	0.83 (0.55, 0.94)*	2.08 ± 1.53
BWS 0° Tilt (*n* = 15)	130 ± 9	130 ± 9	0.89 (0.70, 0.96)*	1.88 ± 1.28
BWS 9.5° HUT (*n* = 15)	130 ± 11	131 ± 12	0.95 (0.85, 0.98)*	1.57 ± 1.42

*Note*: Values are the mean ± SD. Sample size varies owing to missing data.

Abbreviations: CI, confidence interval; CV%, within‐participant coefficient of variation; HDT, head‐down tilt, HUT, head‐up tilt, ICC, intraclass correlation coefficient; LOP, limb occlusion pressure.

*P < 0.0001.

## 4 DISCUSSION

### 4.1 Summary of main findings

The aim of this study was to examine the effect of simulated micro‐ and hypo‐gravity on leg and arm LOP within bedrest and BWS analogues. Short‐term exposure to these settings elicited a small but significant increase in leg LOP at 9.5° HUT by 9–15 mmHg compared with 6° HDT. Leg LOP displayed a significant increase in the BWS analogue compared with bedrest in the 0° tilt and 9.5° HUT conditions by 8 mmHg. Arm LOP remained unaffected across body tilts and between bedrest and BWS analogues. Both leg and arm LOP displayed good‐to‐excellent reliability at all examined body tilt conditions (ICCs ≥ 0.83). These findings suggest that factors independent of weight‐bearing can influence leg LOP within an individual. Application of BFR as a spaceflight countermeasure should, therefore, take this into consideration when developing operational protocols for implementing BFR in real micro‐ and/or hypo‐gravity environments. It would be advisable for astronauts to determine LOP in‐mission before the application of BFR to prescribe tourniquet pressure accurately.

### 4.2 Influence of body tilting on LOP

Previous research identified that LOP differs significantly between lying, sitting and standing positions (Hughes, Jeffries, et al., 2018; Sieljacks et al., 2017). The present findings additionally demonstrate that supine body tilting independent of weight‐bearing also has a small but significant effect on leg LOP by 9–15 mmHg (5%–9%) between 6° HDT and 9.5° HUT. Posture‐related changes in leg LOP have been explained previously by increases in peripheral blood flow and lower extremity venous blood pooling owing to the effects of gravity when transitioning from a supine to a sitting or standing position (Hughes, Jeffries, et al., 2018). In comparison, HDT induces fluid redistributions from the legs to the thorax and head (Tomaselli et al., 1987). Indeed, major cardiovascular parameters (e.g., stroke volume and systemic vascular resistance) exhibit significant sensitivity to head‐down and head‐up manoeuvres whereby the gravitational vector acting along the body is manipulated (Whittle et al., 2022). The present study observed leg LOP to be lowest in the 6° HDT condition and highest at 9.5° HUT, supporting that fluid redistributions and/or altered cardiovascular regulation could be influencing leg LOP during body tilting. Furthermore, the body tilts examined during bedrest were independent of longitudinal (head‐to‐toe) weight‐bearing, suggesting that factors beyond muscle contractile state and limb position influence leg LOP, which prior research examining LOP between lying, sitting and standing has been unable to differentiate between.

Arm LOP did not change significantly across the examined body tilts. Arm LOP has been found previously to be less sensitive to postural changes in comparison to leg LOP. For instance, arm LOP changed by 5%–8% (5–12 mmHg) when transitioning between standing, sitting and supine lying positions, whereas leg LOP changed by 23% (55 mmHg) between lying and standing (Hughes, Jeffries, et al., 2018; Karanasios et al., 2022; Sieljacks et al., 2017). The present findings are in support of this notion through the observation that arm LOP remained unaffected between the examined body tilt conditions, whereas leg LOP was affected.

For application of BFR in bedrest or BWS analogues, one must consider that body tilt can affect leg LOP with even small tilt angles. Given that 6° HDT is an internationally recognized analogue for microgravity and that 9.5° HUT can be used as a lunar gravity analogue, the findings also suggest that LOP, at least in the leg, might differ between reduced gravitational environments. Astronauts using BFR in future exploration missions, therefore, would benefit from in‐mission LOP measures before the application of BFR, in order to prescribe tourniquet pressure accurately. Theoretically speaking, it would seem impractical and time‐consuming for each astronaut to measure LOP via Doppler ultrasound before every exercise session using BFR in‐mission; therefore, the most practical means of measuring LOP would be to use a valid and reliable BFR tourniquet system capable of measuring LOP automatically.

### 4.3 Influence of bodyweight suspension and bedrest analogues on LOP

The present study identified that despite maintaining the same body tilt, a significantly higher pressure was required to occlude the leg in BWS relative to bedrest at 0° tilt and 9.5° HUT. The increase in leg LOP during BWS at 9.5° HUT could be attributed to participants having to stand with ∼20% bodyweight loading, which requires contraction of lower limb anti‐gravity (postural) muscles, whereas participants remained non‐weight‐bearing during 9.5° bedrest. Previous research has established that during a standard HUT procedure to induce orthostatic stress, vertical weight‐bearing causes activation of antigravity muscles (e.g., soleus) alongside altered cardiovascular responses (e.g., higher calf blood flow) relative to when participants were non‐weight‐bearing in the same HUT position (Shamsuzzaman et al., 1998). Interestingly, however, we observed the same increase in leg LOP during BWS in the 0° tilt position relative to bedrest, where participants were non‐weight‐bearing in both cases. Some suggestions regarding why this happened might include the BWS leg slings and back support plate concentrating areas of tissue compression (relative to bedrest), specifically at the thigh and gluteal regions. The perceived sensation of reduced bodyweight, floating and/or instability caused by BWS, potential discomfort owing to BWS, or exposure to a new setting could have also altered factors such as muscle tone and/or cardiovascular regulation (e.g., increased sympathetic activity) relative to bedrest, which might influence LOP. However, because there were no statistical differences in leg LOP during 6° HDT or in arm LOP in any position between bedrest and BWS analogues, the underlying causes for these responses, or lack thereof, remain unclear. To this end, it is recommended that LOP be measured in the setting/analogue to which BFR is subsequently applied, even when the position/tilt of the individual is maintained.

### 4.4 Reliability of LOP during bodyweight suspension and bedrest

A reliable LOP measurement helps to minimize the risk of over‐ or underprescribing tourniquet pressure, which can influence the effectiveness, safety and tolerability of BFR (Patterson et al., 2019). The present study demonstrated that LOP can be measured reliably during BWS and bedrest in 6° HDT, 0° tilt and 9.5° HUT positions in the leg (ICCs ≥ 0.91) and arm (ICCs ≥ 0.83), with minimal within‐participant variation (1%–2%). These findings are comparable to previous studies investigating the effects of body posture (standing, sitting and supine lying) on leg LOP using the Delfi Personalized Tourniquet system (ICCs ≥ 0.95 and CVs of 2%–3%) and for arm LOP measured via Doppler ultrasound (ICCs ≥ 0.86 and CVs of 5%–6%) (Hughes, Jeffries, et al., 2018; Karanasios et al., 2022).

### 4.5 Limitations

The present study sample was composed predominantly of young healthy adults; therefore, generalization of the present findings to other populations should be made with caution. It remains possible that LOP might be more or less sensitive to changes in body tilts between groups based on sex, age and/or health status. Only three body tilts were examined; therefore, we were unable to explore the relationship between LOP and a wide range of body tilts. The LOP was examined after short exposures to the body tilts; however, it is possible that LOP could drift during extended exposures (e.g., >1 h), which could have implications for scenarios in which the body transitions between different HUTs or HDTs or gravitational environments for extended durations. For example, spaceflight causes muscle atrophy, which alters limb circumference and can cause a decline of ≤10 mmHg in systolic blood pressure, with both variables being significant predictors of LOP in a healthy population (Brown et al., 2018; Evin et al., 2021; Loenneke et al., 2015; Norsk, 2020). Although 6° HDT can induce cephalad fluid shifts, this position does not eliminate gravitational loading on the body that would otherwise occur in space, thus creating areas of pressure on the posterior side of the body beneath the plinth/slings and compressing the thorax and cardiac/vascular tissues (Norsk, 2020). Astronauts in space also assume a semi‐crouched position, with the arms partly flexed in front of the body, whereas in the present study, participants remained supine (Han Kim et al., 2019). Furthermore, 6° HDT does not consider other aspects of the spaceflight environment that an astronaut would experience, including the stress of launch, ambient and extreme noises, vibrations or spaceflight‐associated motion sickness.

Simulating surface gravity on the moon using a 9.5° HUT position also has limitations. First, the 9.5° HUT position using a tilting bed/platform alone does not induce any loading at the feet that would otherwise be present on the moon. The use of the BWS, therefore, allowed for a higher‐fidelity simulation by allowing a component of Earth's gravity to act along the longitudinal axis of the body, permitting participants to stand with ∼20% body weight. Importantly, although 9.5° HUT is necessary to mimic the gravitational environment of the lunar surface, it remains to be established whether the 9.5° HUT position mimics the cardiovascular responses to lunar gravity. Some have proposed the use of a 2° HUT or 9.5° HUT with compression stockings to try to replicate estimated plasma volume losses in lunar gravity (Brinley et al., 2009); however, until sufficient data can be obtained from future crewed missions to the moon it remains uncertain how analogues can be modified to replicate both the mechanical and the cardiovascular responses to such an environment.

### 4.6 Future research

Progressing BFR research in a spaceflight context is important to determining its effects within real and simulated micro‐ and hypo‐gravity settings to inform whether it can feasibly be used as a countermeasure in future space exploration missions. Bedrest and BWS serve as two useful analogues to begin initial investigations into whether BFR can be prescribed accurately and reliably in these unique settings, and this study has provided early insights on methodological considerations for use of BFR in these settings (e.g., the effect of body tilt and analogue on LOP). Expanding upon the present investigation regarding whether BFR can be prescribed feasibly and reliably in future mission scenarios would require examination of the effects of real reduced gravitational loading on LOP by means of, for example, parabolic flights (Shelhamer, 2016). To progress understanding of how LOP responds to various conditions, it would be valuable to characterize the relationship between LOP and body tilt at a wider range of angles (e.g., ±45°) to examine whether it follows a dose–response relationship that the present findings suggest might be occurring, at least in the leg. Furthermore, it has not yet been established what causes (or is associated with) acute posturally related changes in LOP that would otherwise help to improve our general understanding of why LOP is sensitive to body tilting/postural changes and why the responses are more pronounced in the leg than in the arm (Hughes, Jeffries, et al., 2018; Karanasios et al., 2022).

New evidence suggests that improvements in maximal aerobic capacity following BFR aerobic training might be haematologically mediated (Thompson et al., 2023). During treadmill walking with BFR, a considerable release of blood volume‐regulating hormones (renin and copeptin) has been reported, perhaps owing to the sequestering of a large blood volume away from the central circulation, which could potentially induce hypervolaemia and erythropoiesis after multiple training sessions (Thompson et al., 2023). Given the significant impact of gravity on the cardiovascular system (e.g., central/headward fluid shifts), among other systems, it seems prudent for future research to examine whether BFR training in reduced gravity environments induces similar acute/chronic physiological responses via mechanisms comparable to those reported in terrestrial settings and the implications if such responses are found to differ (Norsk, 2020).

## 5 CONCLUSION

The present study demonstrated that LOP at the leg exhibited a small but significant increase from 6° HDT to 9.5° HUT by 9–15 mmHg (5%–9%) during bedrest and BWS, whereas arm LOP remained unaffected. This suggests that factors beyond muscle contractile state and limb posture influence LOP. In addition, leg LOP differed between bedrest and BWS analogues in the same body position for two of the three examined body tilts, suggesting that the setting in which LOP is measured (independent of posture) might also be important to consider. It is recommended that LOP be measured at the same body tilt and setting to which the vascular occlusion is subsequently applied to minimize any discrepancies between the actual and target tourniquet pressure. Micro‐ and hypo‐gravity environments might impact the acute/chronic physiological responses to BFR training relative to Earth. Future research is warranted to examine the effects of BFR exercise in reduced gravity settings and the implications of BFR as a spaceflight countermeasure.

## AUTHOR CONTRIBUTIONS

The study was conducted in the Aerospace Medicine and Rehabilitation Laboratory (Northumbria University, UK). All authors contributed to: (1) the conception or design of the work; (2) the acquisition, analysis or interpretation of data for the work; and (3) drafting the work or revising it critically for important intellectual content. All authors approved the final version of the manuscript and agree to be accountable for all aspects of the work in ensuring that questions related to the accuracy or integrity of any part of the work are appropriately investigated and resolved. All persons designated as authors qualify for authorship, and all those who qualify for authorship are listed.

## CONFLICT OF INTEREST

Luke Hughes serves in a scientific and educational capacity for the manufacturer of the personalized tourniquet system used in the present study.

## FUNDING INFORMATION

None.

## Data Availability

Data pertaining to this study have been made publicly available in the open‐access Zenodo repository and can be found at https://doi.org/10.5281/zenodo.10796514.
